# One Signal, Multiple Pathways: Diversity Comes from the Receptor

**DOI:** 10.1371/journal.pbio.0040131

**Published:** 2006-04-04

**Authors:** Liza Gross

Type “Wnt” into Google Scholar, and you'll get nearly 72,000 hits, revealing the pivotal role this widely conserved family of signaling proteins plays in development and disease. Wnt proteins trigger complex signaling cascades that regulate cell growth, migration, differentiation, and many other aspects of development with the help of numerous interacting components. In the best-understood, “canonical” pathway, Wnt signaling molecules (called ligands) bind simultaneously to two coreceptors on the cell surface (Frizzled and LRP), allowing β-catenin proteins to stabilize (avoid destruction), enter the nucleus, associate with the transcription factor complex TCF/LEF, and activate genes involved in cell survival, proliferation, or differentiation. Inappropriate activation of β-catenin has been linked to several types of cancer.

Wnt ligands have also been implicated in several alternative, “noncanonical” pathways, challenging researchers to figure out how proteins that appear so similar at the sequence level can produce such different results. Studies in frogs and zebrafish embryos suggest this diversity derives from engaging multiple pathways, with Wnt5a, for example, triggering an intracellular calcium release that activates calcium-dependent signaling molecules. It's also possible that Wnt5a signals through other receptors (besides the canonical Frizzled receptor) with a Wnt-binding domain, such as the receptor tyrosine kinase-like orphan receptor 2 (Ror2). But because isolating Wnt ligands in a soluble form has proven difficult, scientists have been forced to resort to indirect methods of studying the mechanisms of Wnt studies, which often provided varying and conflicting results.

In a new study, Amanda Mikels and Roel Nusse have developed a technique to purify the Wnt5a protein and directly investigate its contribution to different pathways. They show that soluble Wnt5a proteins can both inhibit and activate the canonical pathway, depending on which combination of receptors is expressed on the cell surface. When Wnt5a interacts with Ror2, the canonical Wnt/β-catenin pathway is inhibited; when it engages Frizzled and LRP, the β-catenin pathway is activated.

The researchers modified a Wnt purification technique previously established in their lab to harvest Wnt5a proteins from cells engineered to overexpress the mouse *Wnt5a* gene, and confirmed the identity of the protein by examining a key part of its amino acid sequence. Having confirmed the identity of the protein, they compared Wnt5a's capacity to mediate signaling in cells expressing different combinations of the Ror2, Frizzled, and LRP surface receptors. They also examined Wnt5a's capacity to modulate signaling by Wnt3a, which is known to activate the canonical pathway.

First, the researchers tested the possibility that Wnt5a could also activate β-catenin signaling in a cultured cell line (called 293 cells) and found that it could not. But when they treated cells with both Wnt3a and Wnt5a, they discovered that Wnt5a could prevent activation of the β-catenin-dependent TCF transcription factor by Wnt3a. It was initially unclear how this happened. Wnt5a could compete with Wnt3a for the Frizzled receptor, or it might activate a gene that targets β-catenin for destruction. Either way, β-catenin levels should drop following treatment with Wnt5a. Yet β-catenin levels were unaffected; furthermore, Wnt5a didn't interfere with β-catenin's entry into the nucleus. These results indicate that Wnt5a did not block Wnt3a signaling through either of these routes. The researchers also show that Wnt5a doesn't rely on calcium-dependent signals, as had been suggested in previous work. Thus, Wnt5a must act through some other pathway to block β-catenin signaling by canonical Wnts such as Wnt3a.

Previous studies had suggested that Wnt5a might be able to bind another cell-surface receptor, Ror2, based on evidence that blocking expression of either Wnt5a or Ror2 produces the same effects in animals. And this line of investigation proved fruitful: Mikels and Nusse found that Ror2 is needed for Wnt5a-mediated repression of canonical β-catenin signaling. Additionally, by creating multiple Ror2 constructs lacking different combinations of their binding domains, they showed that Wnt5a binding triggers Ror2-mediated signaling inside the cell.

Interestingly, under very specific conditions—when the coreceptors Frizzled 4 (Frz4) and LRP5 are present—Wnt5a can actually trigger β-catenin accumulation and activate canonical β-catenin gene targets. Since 293 cells do not normally express Frz4, but do express Ror2, the predominant signal prompted by Wnt5a in these cells is inhibition of β-catenin signaling—indicating that different combinations of cell-surface receptors drive different signaling outcomes for Wnt5a.

Whether Wnt5a inhibits β-catenin signaling—performing its job as a tumor suppressor—or activates β-catenin's cell growth and proliferation targets—setting the stage for tumor formation—depends on which receptors are present on the surface of the cell in question. The next challenge will be to identify the mechanism through which Wnt5a blocks the β-catenin pathway. By showing that one Wnt ligand can function through two separate pathways, Mikels and Nusse have opened the floodgates for researching the possibility of dual functionality in the 19 mammalian homologs identified so far. This ability to stimulate different receptors with distinct results is unique for Wnt proteins, but it has been documented in other systems and may well represent an alternate strategy for effecting flexible responses under changing conditions.

## 

**Figure:**
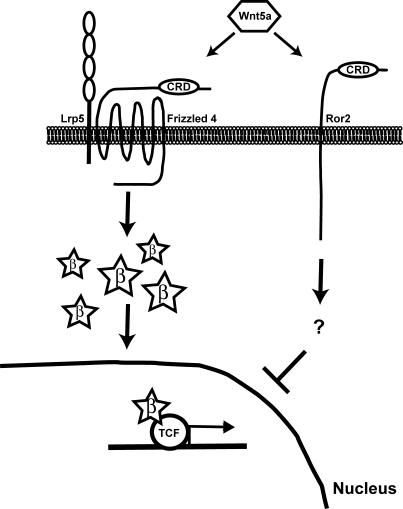
The Wnt5a protein can either activate or inhibit canonical Wnt signaling depending on which receptors are expressed. (CRD stands for the extracellular cysteine-rich Wnt binding domain present in the Fz4 and Ror2 receptors.)

